# P-2292. The Usual Suspect? Prevalence of *Pseudomonas aeruginosa* in Diabetic Foot Infections among Kidney Transplant Recipients

**DOI:** 10.1093/ofid/ofae631.2445

**Published:** 2025-01-29

**Authors:** Margaret E McCort, Rohan Goyal, Daniel A Burack, Phyu Thwe, Luz Liriano Ward, Rachel Bartash

**Affiliations:** Montefiore Medical Center / Albert Einstein College of Medicine, Bronx, New York; Albert Einstein College of Medicine/Montefiore Medical Center, Bronx, New York; Montefiore Medical Center, New York, New York; Montefiore Medical Center, New York, New York; Albert Einstein College of Medicine/Montefiore Medical Center, Bronx, New York; Montefiore Medical Center, New York, New York

## Abstract

**Background:**

Diabetic foot infections (DFIs) are a significant cause of morbidity in kidney transplant recipients (KTRs) with underlying diabetes. While empiric antibiotic coverage with an antipseudomonal antibiotic is common practice, recent studies of DFIs in non-transplant patients have found a relatively low prevalence of *Pseudomonas aeruginosa (*PSA*)*. We hypothesize that the prevalence of PSA in DFIs is also low among KTRs.

Organisms isolated in cultures from diabetic foot infections (DFI) among kidney transplant recipients (KTR)

**Figure:**
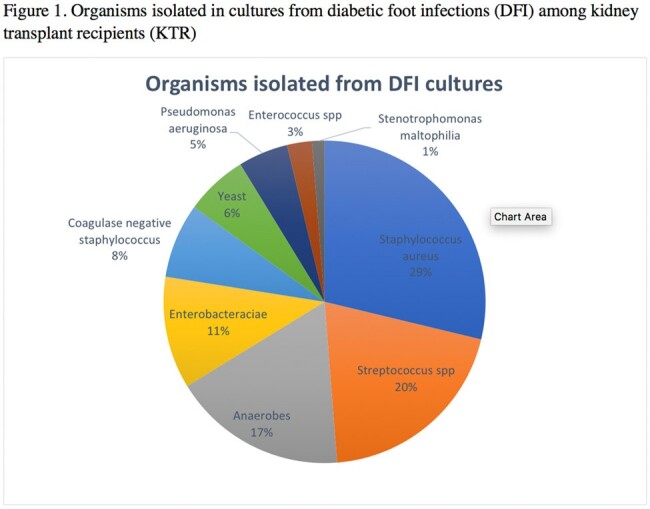

**Methods:**

We performed a review of all positive microbiologic isolates from clinically relevant DFIs in KTRs requiring hospitalization at an urban transplant center from 1/2018-12/2023. All patients were > 18 years of age and underwent renal transplantation between 2015-2023. Patients could be included for more than 1 DFI if these occurred > 6 months apart or on non-contiguous sites. Patients without cultures or with cultures that yielded negative results were excluded. The primary outcome was the prevalence of PSA in microbiologic cultures, including bone, tissue, and fluid sources. The study was approved by our Institutional Review Board and results were reported using descriptive statistics.

Patient characteristics and demographics

**Figure:**
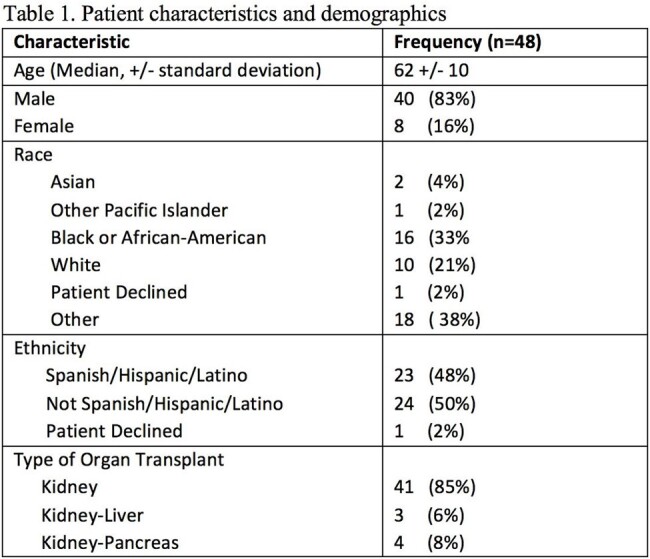

**Results:**

Forty-eight DFIs occurring in 40 KTRs were included. Median age was 62 and 83% (40/48) occurred in men. Average time from transplant to DFI was 1075.5 days. Additional demographic data is shown in Table 1. Eighty organisms were isolated with 42% (20/48) of infections being polymicrobial. PSA represented 5% of organisms isolated (4/80) and was present in cultures from 4 unique patients (10%). Streptococcus species and *Staphylococcus aureus* were the most commonly isolated organisms, representing 20% (16/80) and 29% (23/80), respectively. Among *Staphylococcus aureus* isolates, 47% were methicillin-resistant. Additional microbiologic data is shown in Figure 1.

**Conclusion:**

The prevalence of PSA in DFIs was low among KTRs. Typical skin pathogens including Streptococcus species and Staphylococcus aureus were most common. While larger studies are needed to confirm these results, judicious use of antipseudomonal antibiotics should be considered in DFI management.

**Disclosures:**

All Authors: No reported disclosures

